# A photonic crystal material for the online detection of nonpolar hydrocarbon vapors

**DOI:** 10.3762/bjnano.13.9

**Published:** 2022-01-25

**Authors:** Evgenii Sergeevich Bolshakov, Aleksander Vadimovich Ivanov, Andrei Arkad’evich Kozlov, Anton Sergeevich Aksenov, Elena Vladimirovna Isanbaeva, Sergei Evgen’evich Kushnir, Aleksei Dmitrievich Yapryntsev, Aleksander Evgen’evich Baranchikov, Yury Aleksandrovich Zolotov

**Affiliations:** 1 Department of Chemistry, Lomonosov Moscow State University, Moscow, 119991, Russia; 2 Kurnakov Institute of General and Inorganic Chemistry, Russian Academy of Sciences, Moscow, 119991, Russia; 3 Institute of Fine Chemical Technologies, Russian Technological University Moscow, 119454, Russia; 4 Department of Materials Science, Lomonosov Moscow State University, Moscow, 119991, Russia

**Keywords:** diffuse reflectance spectroscopy, photonic crystal sensor, stimuli-responsive materials

## Abstract

A modern level of nanotechnology allows us to create conceptually new test systems for chemical analyses and to develop sensitive and compact sensors for various types of substances. However, at present, there are very few commercially available compact sensors for the determination of toxic and carcinogenic substances, such as organic solvents that are used in some construction materials. This article contains an overview of how 3D photonic crystals are used for the creation of a new test system for nonpolar organic solvents. The morphology and structural parameters of the photonic crystals, based upon a crystalline colloidal array with a sensing matrix of polydimethylsiloxane, have been determined by using scanning electron microscopy and by the results of specular reflectance spectroscopy based on the Bragg–Snell law. A new approach has been proposed for the application of this sensor in chemical analysis for the qualitative detection of saturated vapors of volatile organic compounds due to configuration changes of the photonic bandgap, recorded by diffuse reflectance spectroscopy. The exposure of the sensor to aromatic (benzene, toluene and *p*-xylene) and aliphatic (*n*-pentane, *n*-heptane, *n*-octane and *n*-decane) hydrocarbons has been analyzed. The reconstitution of spectral parameters of the sensor during the periodic detection of saturated vapors of toluene has been evaluated.

## Introduction

Photonic crystals (PhCs) used for chemical sensors can be divided into three groups depending upon their structure, that is, one-dimensional (1D), two-dimensional (2D), and three-dimensional (3D) [1–10]. 2D and 3D structures used as chemical sensors are studied in most projects. 2D structures consist of a monolayer of spherical particles placed on a substrate. 3D structures, which appear in the form of a crystalline colloidal array (CCA), are called opal structures (spherical particles close-packed in an ordered structure). If the structure has been placed in a matrix and the particles have been removed, then it is an inverse opal structure [11–13]. A photonic bandgap (PBG) appears in colloidal crystals due to the periodic modulation of the refractive index. At the bandgap, selective reflection of light is observed, which is connected to a low photon density of states within the materials [14]. Most of the configuration changes of the photonic bandgap in opal and inverse opal structures occur due to swelling or compression of the polymer matrix or gel.

To date, four main methods for the modification of photonic crystals are used for the creation of stimuli-responsive materials: (a) formation of a sensitive polymer matrix, (b) impregnation of the reagent, (c) immobilization of the reagent and (d) preparation of the sensor elements from molecularly imprinted polymers.

Organic solvents are usually detected by using polymer matrix sensors (Table 1) through matrix interaction [7–9,15–16]. The impregnation and immobilization methods are rather close; they are used for the determination of inorganic ions (Cu^2+^, Pb^2+^, Hg^2+^, Ni^2+^ and Cd^2+^) [2,11,17–19] and organic molecules of simple and complex structure (glucose, organophosphates, urea, creatinine, ciprofloxacin and sarin) [5–6,20–27]. The development of a sensor device with molecularly imprinted polymers allows for the determination of organic compounds (nicotinamide, sulfonamides, bisphenol A and diethylstilbestrol) with a more complex structure [12–13,28–29].

**Table 1:** Some photonic crystal structures for chemical sensing of organic solvents.

Geometry	Material	Response	Analyte	Limit of detection	Ref.

3D PhC (CCA)	polystyrene	redshift (<50 nm)	methanol vapors	5% (*V*_methane_/*V*_0_)	[7]
3D PhC (PCCA)^a^	polystyrene-Ag/ polydimethylsiloxane	redshift (<50 nm)	chloroform, chlorobenzene, tetrahydrofuran, dichloromethane and dimethoxyethane liquids	5 µL (5 nm)	[8]
3D PhC (CCA)	polystyrene	redshift (<40 nm)	methanol, ethanol, isopropanol, 1-propanol and *n*-butanol vapors	2% (*V*_ethane_/*V*)	[9]
3D PhC (PCCA)	polymethylmethacrylate/ methyl cellulose	redshift (<80 nm)	ethanol, *n*-propanol, isopropanol and *n*-butanol liquids and vapors	NA	[15]
3D PhC (CCA)	polystyrene	redshift	methanol and ethanol	NA	[16]
3D PhC (PCCA)	polystyrene/ polydimethylsiloxane	redshift (<150 nm)	benzene, toluene, *p*-xylene, *n*-pentane, *n*-heptane, *n*-octane and *n*-decane vapors	0.3 mg/m^3^ (toluene)	this work

^a^Polymerized crystalline colloidal array.

The color shift (blueshift or redshift) or the color intensity of the sensor serves as an analytical signal for such sensors. The standard method for measuring an analytical signal is specular reflectance spectroscopy within the visible range; however, the interaction of a 2D PhC with the analyte is also analyzed by changing the diameter of the Debye diffraction ring [2,5–6,10].

Currently, very few works are devoted to the study of the mechanism that leads to the shift of the PBG in 2D and 3D photonic crystals. This is caused by the variety of flow processes in the structure, which are significantly influenced by the filling of the structure, the structural heterogeneity within a volume, the presence of foreign chemical substances and the size variation of the matrix and particles during the chemical analysis process. However, one cannot ignore sensors based on molecularly imprinted polymers for the selective detection of volatile organic compounds [30–32]. In most cases, the response of such sensors is a change in mass recorded using a quartz microbalance. A simpler design and research method made it possible to investigate in more detail the processes occurring during the absorption of solvents. The ability to control selectivity in molecularly imprinted sensors and the simple visual response in photonic crystal sensors make it promising and even mandatory to combine these two approaches.

In our previous study, the optimal parameters of polystyrene (PS) particles for sensor matrices for saturated vapors of volatile organic compounds have been determined [33]. In this work, we determined the parameters of the sensor structure and examined online the detection of high concentrations of aromatic and aliphatic hydrocarbon vapors in air. The detection was performed by using 3D PhC-based sensors, which are a CCA of polystyrene submicrometer particles embedded in a polydimethylsiloxane (PDMS) layer. The matrix interaction was responsible for the main mechanism, which was monitored by configuration changes of the photonic bandgap using diffuse reflectance spectroscopy.

## Results and Discussion

### Determination of the morphology and the structural parameters of the sensor

**Figure 1:**
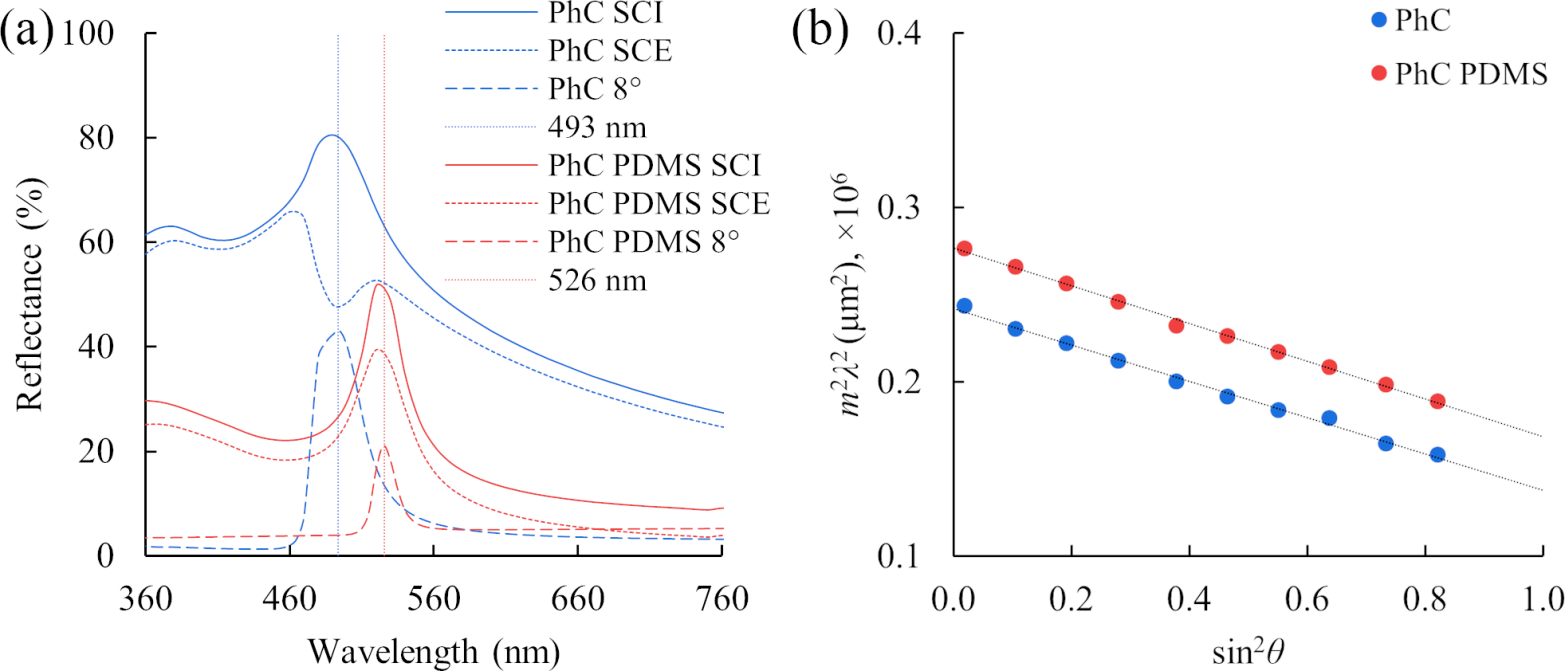
Optical characteristics of sensors based on 3D PhC (matrix thickness ≈ 90 µm): (a) diffuse reflectance and specular (8°) reflectance spectra of the sensor before and after the PDMS layer formation (the difference in reflection intensity is caused by different apertures and the area of samples) and (b) dependence of *m*^2^λ^2^ on sin^2^θ for the sensor with the PDMS layer (red circles) and without (blue circles), where *m* is the order of the photonic bandgap, λ is the wavelength of the photonic bandgap, θ is the incident angle. Black lines show the linear correspondence of the experimental data.

A comparison between the specular reflectance and the diffuse reflectance spectra tested in the “specular component included (SCI)” and “specular component excluded (SCE)” modes has shown (Figure 1a) that the maximum of the diffuse reflectance spectra of the PhC sensor in the SCI mode coincides with the maximum of the specular reflection spectra resulting from flat (111) surfaces at an 8° angle. This applies regardless whether the sensor has a PDMS layer or not. However, the diffuse reflectance spectra of the sample without a polydimethylsiloxane layer, measured in the SCE mode, had a significant intensity decrease at the assumed maximum point of the reflection. For a sensor with a PDMS layer, the spectrum intensity of the specular reflectance component, regarding the diffuse reflectance spectrum, decreases. This is expressed in the smaller influence of the viewing angle on the color of the sensor and a simpler visual registration of the color due to a decrease of the iridescence effect, which is an important requirement for the testing system.

A reflection peak was approximated by a quadratic function (*y* = *ax*^2^ + *bx* + *c*) to determine the PBG position. The fitted coefficients of the quadratic function of spectral maxima were used to determine the dependence of the photonic bandgap position on the exposure time and the incident angle by the analysis of the diffuse and specular reflectance spectra.

**Figure 2:**
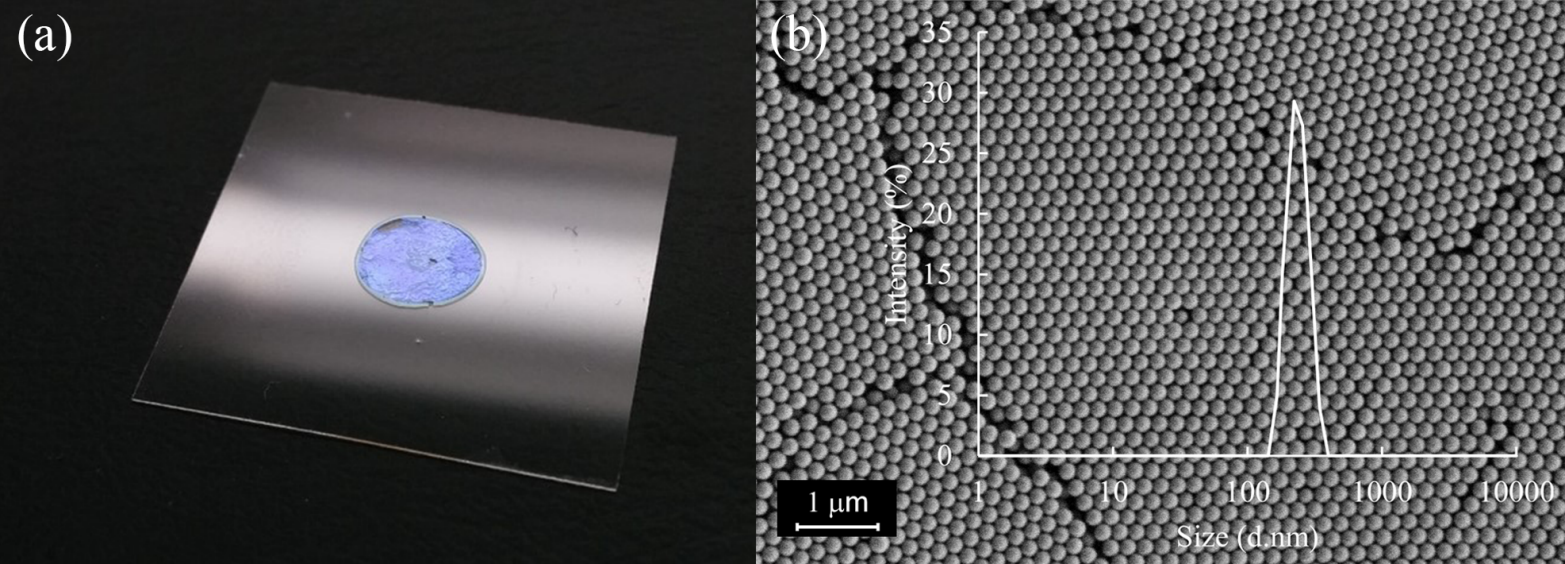
(a) Photo of the sensor and (b) electron microscopy image of a CCA of polystyrene particles without a PDMS layer on a glass substrate (effective particle size = 201 nm). The data obtained with dynamic light scattering slightly overestimates the diameter in comparison with the data obtained from microphotographs. This may be related to the specificity of the dynamic light scattering (DLS) method (hydrodynamic diameter measurement).

According to the literature data, the structure has a face-centered cubic lattice (FCC), therefore, it can be assumed that the structure is filled by 74% with PS and that the remaining volume is filled with air or polydimethylsiloxane [34–35]. Figure 2 shows the surface of a crystalline colloidal array obtained by using scanning electron microscopy.

The investigated samples have an ordered lattice structure similar to crystals; therefore, the Bragg equation has been applied for the analysis. Since the diameter of particles in the lattice is in the submicrometer region, diffraction occurs in the visible spectrum, and it becomes necessary to consider the refraction of light during propagation through materials with different refractive indices. In the case of the CCA with FCC lattice, the Bragg–Snell law can be written as follows:


[1] $$m\lambda =2d_{111}\sqrt{n_{\text{eff}}^{2}-n_{\text{air}}^{2}\sin^{2}\theta },$$

where *m* is the order of a diffraction maximum, λ is the wavelength of the reflectance maximum, *d*_111_ is the interplanar distance between crystallographic (111) planes, *n*_eff_ is the effective refractive index of the CCA, *n*_air_ is the refractive index of the medium (in our case air) from which light falls in and θ is the incidence angle [8–9,36]. The incidence angle was varied from 8° to 65° in the experiments.

Knowing the volume fraction of particles in the CCA (*f*_p_) and their refractive index (*n*_p_), as well as the volume fraction of air or other substances (*f*_f_) that fill the spaces between the particles and the corresponding refractive index (*n*_f_), an effective refractive index of the CCA can be calculated by using the following formula:


[2] $$n_{\text{eff}}^{2}=\sqrt{f_{\text{p}}n_{\text{p}}^{2}+f_{\text{f}}n_{\text{f}}^{2}}.$$

The interplanar distance between crystallographic planes (111) in a FCC lattice is related to the effective particle diameter *D* by the following equation:


[3] $$d_{111}=\sqrt{2/3}D.$$

From the linear dependence (Figure 1b) and Equations 1–3, an average diameter of spherical particles and the effective refractive index of the photonic crystal can be obtained [37–38]. The effective refractive index of the structure without PDMS is 1.525, with PDMS it is 1.599, and the average diameter of the particles is 197.5 nm and 201.5 nm, respectively. The obtained average diameters of the polystyrene particles are consistent with the SEM results, and the effective refractive indices are slightly overestimated in comparison with the theoretically calculated values (without PDMS: 1.477, with PDMS: 1.568) [39–40]. This may be due to the presence of surfactants in the interparticle space, defects of the crystal lattice, and a different ratio of volume fractions.

The use of diffuse reflectance spectrometry in further experiments is necessary to obtain integrated optical characteristics of the stimuli-responsive matrix as an analogue of visual recordings. This allows one to optimize the development of sensors with a visual recording of analysis results.

### Kinetics of interaction between the sensor and solvent vapors

Of interest is the use of a 3D PhC-based sensor for online measurements of the concentration of nonpolar solvents and their vapors. Exposure to saturated solvent vapors allows the analyte to be delivered more evenly to the surface of the stimuli-responsive matrix than applying a liquid sample to the surface of the sensitive layer, which is an important feature in studying the mechanism of interaction. The following analytes were studied: benzene, toluene, *p*-xylene (the BTX aromatics), *n*-pentane, *n*-heptane, *n*-octane and *n*-decane, which have a high vapor pressure under normal conditions.

**Figure 3:**
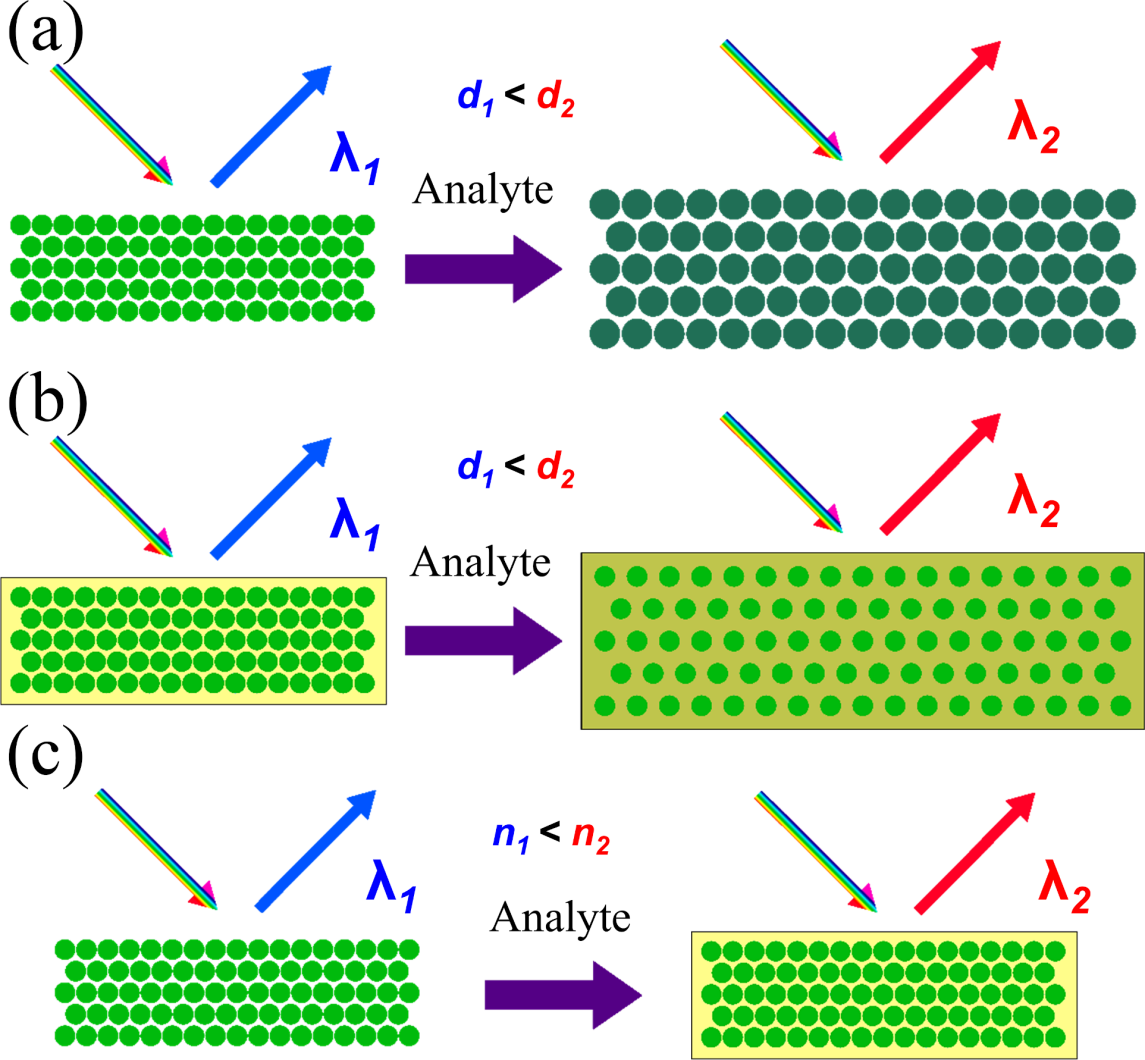
A mechanism of detecting hydrocarbons with a sensor based on a 3D PhC: (a, b) swelling of colloidal particles or polymer matrix, leading to a change in the lattice period *d*; (c) change in the average refractive index of a periodic structure *n*.

As a result of exposure to aromatic and aliphatic nonpolar solvents, a redshift in the PBG is observed using diffuse reflectance spectroscopy. The diffusion of vapors of organic solvents into the PDMS layer and the CCA leads to their swelling and, to a lesser extent, to a change in the effective refractive index of the structure (Figure 3). Since the experimental observation of the photonic bandgap shift was more than 100 nm, this would require a very large change in the effective refractive index. Consequently, the degree of swelling of polydimethylsiloxane predominantly affects the formation of a response when exposed to hydrocarbons. In this regard, it can be assumed that the sensor will be able to detect other hydrocarbons leading to a swelling of PDMS, such as diethyl ether, tetrahydrofuran and chlorobenzene [41]. Therefore, the main factor can be considered the affinity of solvents to PDMS, namely the polarity and rate of diffusion of the solvent through the matrix. In addition, the polydimethylsiloxane matrix protects the polystyrene CCA from the effects of high concentrations of hydrocarbons, thus, it acts as a “conductor” and “dispenser” of the analyte.

**Figure 4:**
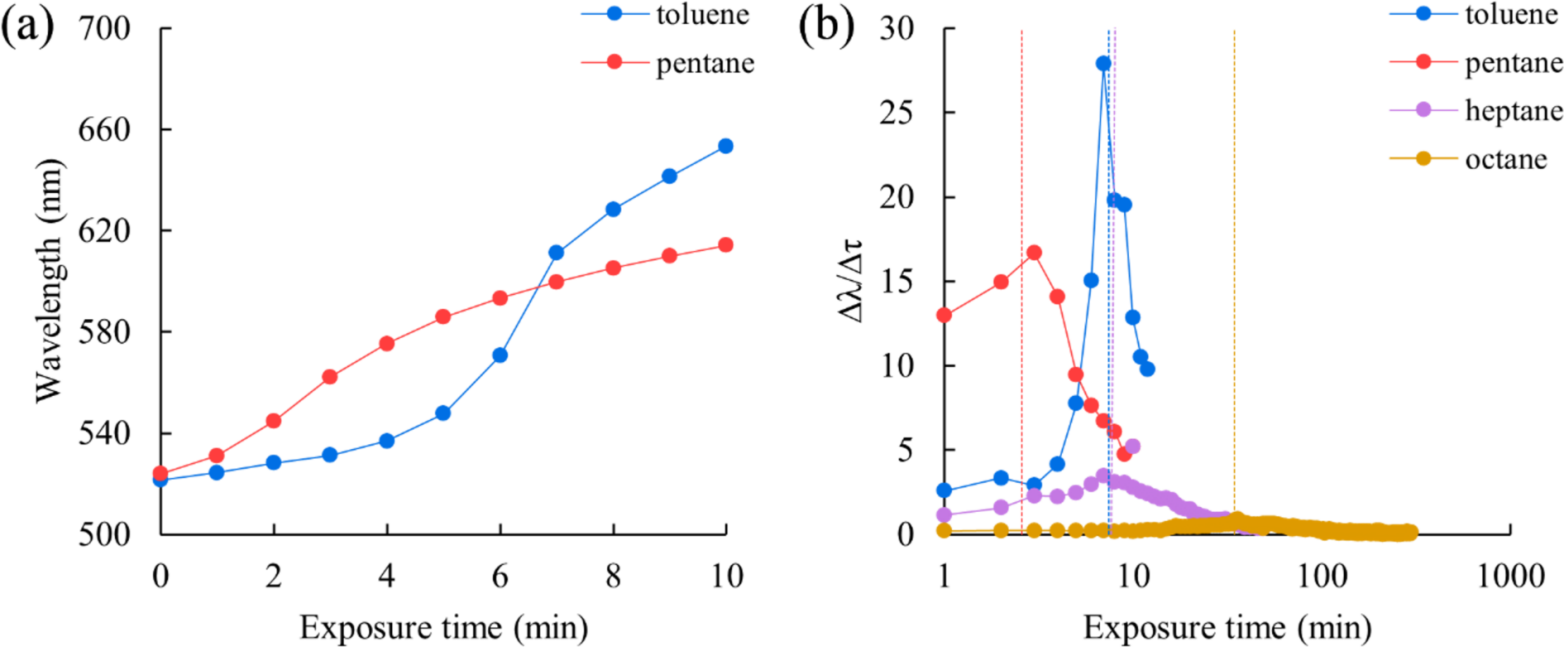
Comparison of the PBG shift rate exposed to toluene and *n*-pentane vapors (matrix thickness about 90 μm): (a) an example of kinetic curves (the experimental temperature was maintained at 23.1–23.6 °C) and (b) differential curves plotted from the average experimental data (additional curves are given for *n*-heptane and *n*-octane).

An analysis of the vapor effects of the analytes was performed through studying kinetic curves (Figure 4a). It was found that nonpolar aromatic compounds have some response delay, but a steeper rise of the S-curve, which is visually expressed as a more contrasting color change of the sensor matrix. The effect of vapors of nonpolar aliphatic organic solvents, in contrast, leads to an instantaneous photonic bandgap shift, but there is no sharp jump in the kinetic curve. The response time means the moment when the PBG shift rate is maximum; this parameter is well determined by the first derivative of the curves from Figure 4b. This feature allows one to distinguish qualitatively the analytes. This behavior can be explained by the rapid dissolution of the necks (“bridges”) between neighboring particles that occur during CCA assembly and hold this array, preventing it from moving apart due to the swelling of the polydimethylsiloxane layer, whereas when exposed to the test alkanes, this does not happen so quickly, and the shift of the lower layers is delayed, resulting in broadening of the photonic bandgap and a less pronounced color.

### Qualitative detection of nonpolar low-molecular-weight organic compounds

**Figure 5:**
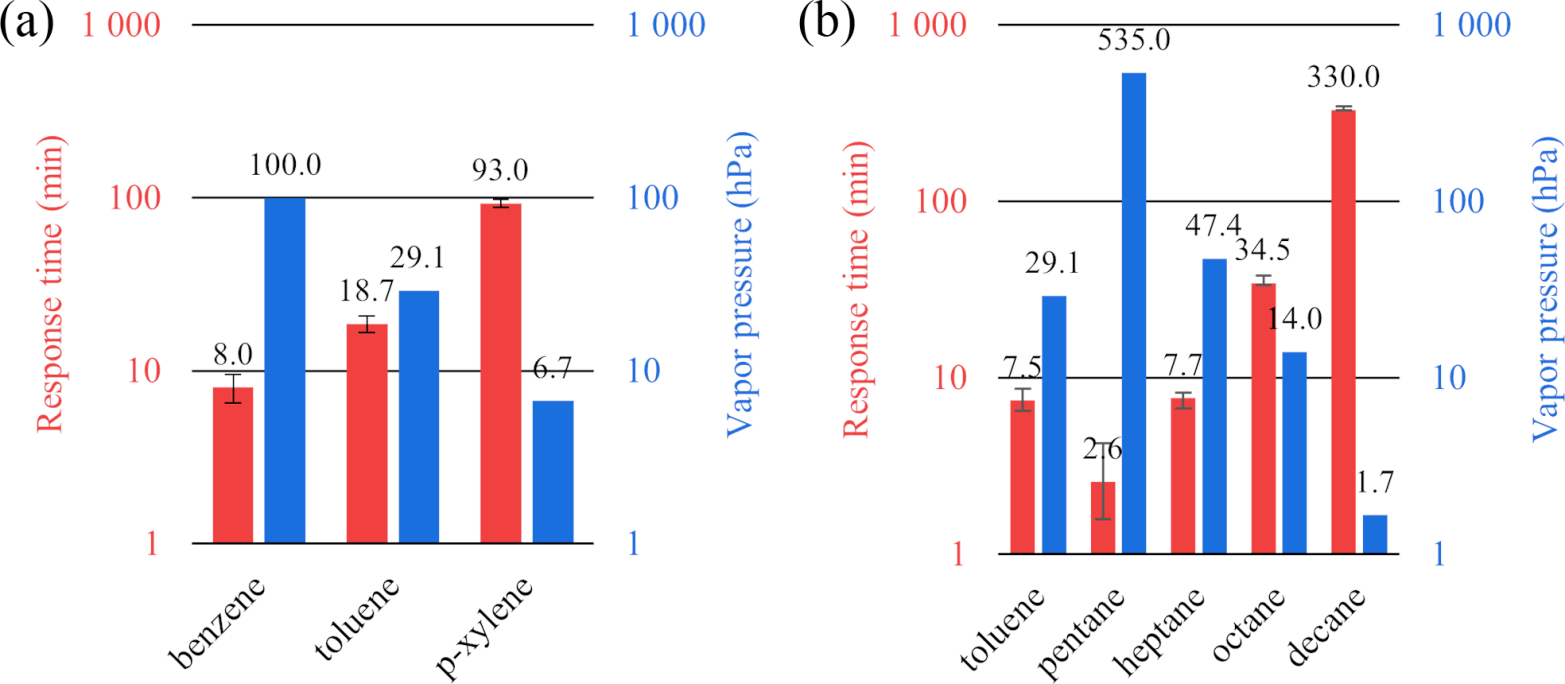
Response rates of sensor matrices (red) and vapor pressure (blue): (a) response time for aromatic hydrocarbons (matrix thickness about 280 μm) and (b) response time for normal alkanes (matrix thickness about 90 μm).

It was found that the response rate increases exponentially among *n*-pentane, *n*-heptane, *n*-octane and *n*-decane. This is consistent with an exponential decrease in vapor pressure and a decrease in the rate of diffusion of the compounds into the polymer matrix (Figure 5). The effect of *n*-decane vapor does not lead to a significant change in the sensor color, but only shifts the PBG by just ≈10 nm, nevertheless, the approach proposed here allows one to detect slight changes in the sensor color.

Aromatic and aliphatic hydrocarbons were screened for the color change time of the sensor. For the series benzene, toluene, *p*-xylene and *n*-pentane, *n*-heptane, *n*-octane and *n*-decane, an increase in the response time is observed that is close to exponential. This allows for the detection of the total toxic effect considering the different analytical response rates and the toxicity of the detected compounds.

Since the absorption of hydrocarbon vapors is responsible for the delivery of the analyte to the photonic crystal, a change in the sensor sensitivity is possible by varying the thickness of the sensitive PDMS layer. In the experiments, sensors with a polydimethylsiloxane layer thickness from 10 μm to 2 mm were investigated for detecting low concentrations of vapors and liquid hydrocarbons. As the limit of detection depends on the matrix thickness, the main obstacle to its reduction is the development of a technique for uniform application of polydimethylsiloxane. In some experiments, it was possible to detect toluene vapors with a concentration of ca. 0.3 mg/m^3^ using a sensor with a sensitive layer thickness about 20 μm.

### Effects of organic solvent mixtures on the sensor

**Figure 6:**
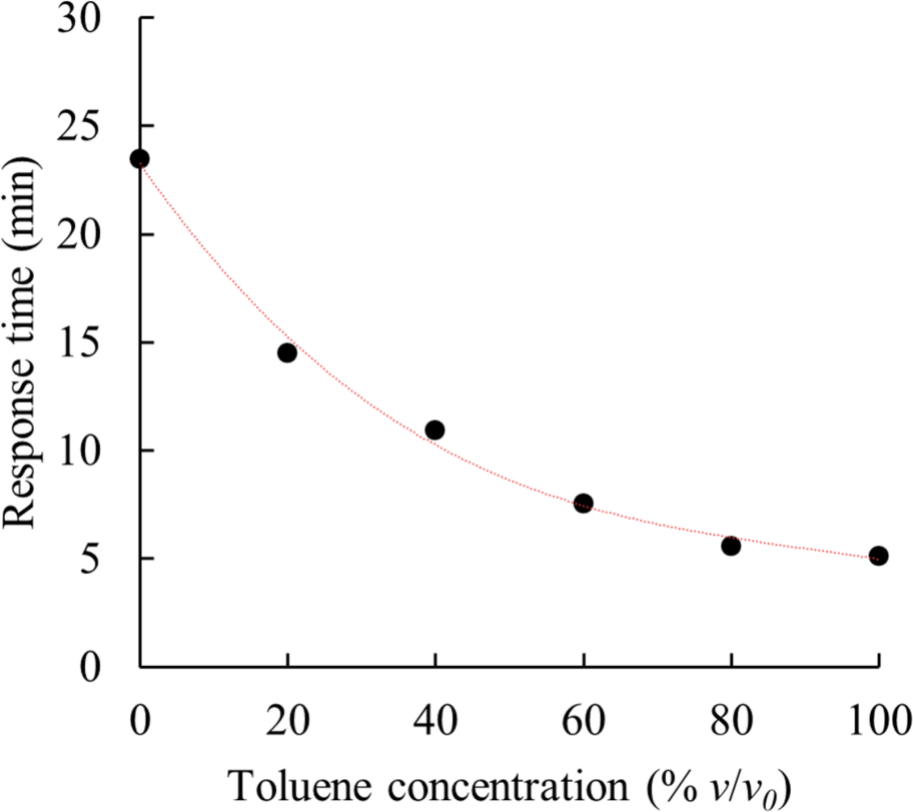
The dependence of the sensor response rate on the content of toluene in *p*-xylene (matrix thickness about 100 μm).

Of particular interest is the detection of analytes in complex objects. An example is the detection of toluene in the presence of xylenes. We discovered that the response rate of the composite sensor is affected by the *p*-xylene/toluene ratio in the analyzed mixture. The relationship shown in Figure 6, correlates well (*R*^2^ = 0.995) with a 3rd-degree polynomial curve: *t* = – 2.0 × 10^−5^*C*_PhMe_^3^ + 4.8 × 10^−3^*C*_PhMe_^2^ – 0.49*C*_PhMe_ + 23.

The rate of sensor response to the mixture increases sharply even with a low content of toluene. This factor indicates the possibility of detecting small concentrations of volatile organic compounds with a higher vapor pressure in complex objects. The experimental results also show the possibility of using a sensor to assess the total toxic effect of BTX vapors.

This approach shows that this sensor can be used for the qualitative analysis of complex matrices such as fossil fuel due to the different types of kinetic curves, for example, as shown for aliphatic and aromatic hydrocarbons, according to the criteria obtained after processing the kinetic curves using chemometric methods of analysis.

### Reversibility of optical characteristics of the sensor during cyclic exposure to toluene

After the first cycle of exposure to both aromatic and aliphatic solvents, the initial color of the sensor changes, associated with the degradation of the CCA. It should be noted that benzene, toluene and *p*-xylene, unlike, for example, *n*-pentane or *n*-heptane, can lead to the irreversible destruction of the stimuli-responsive matrix due to dissolution or adhesion of PS particles. Therefore, an experiment was carried out with cyclic exposure to toluene vapor. The sensor matrix was exposed to saturated toluene vapors for 8 min. Then, the sensor was allowed to stand for a day to recover the photonic bandgap to its initial position, although 90% of recovery was reached already after 2 min, and the experiment was repeated.

**Figure 7:**
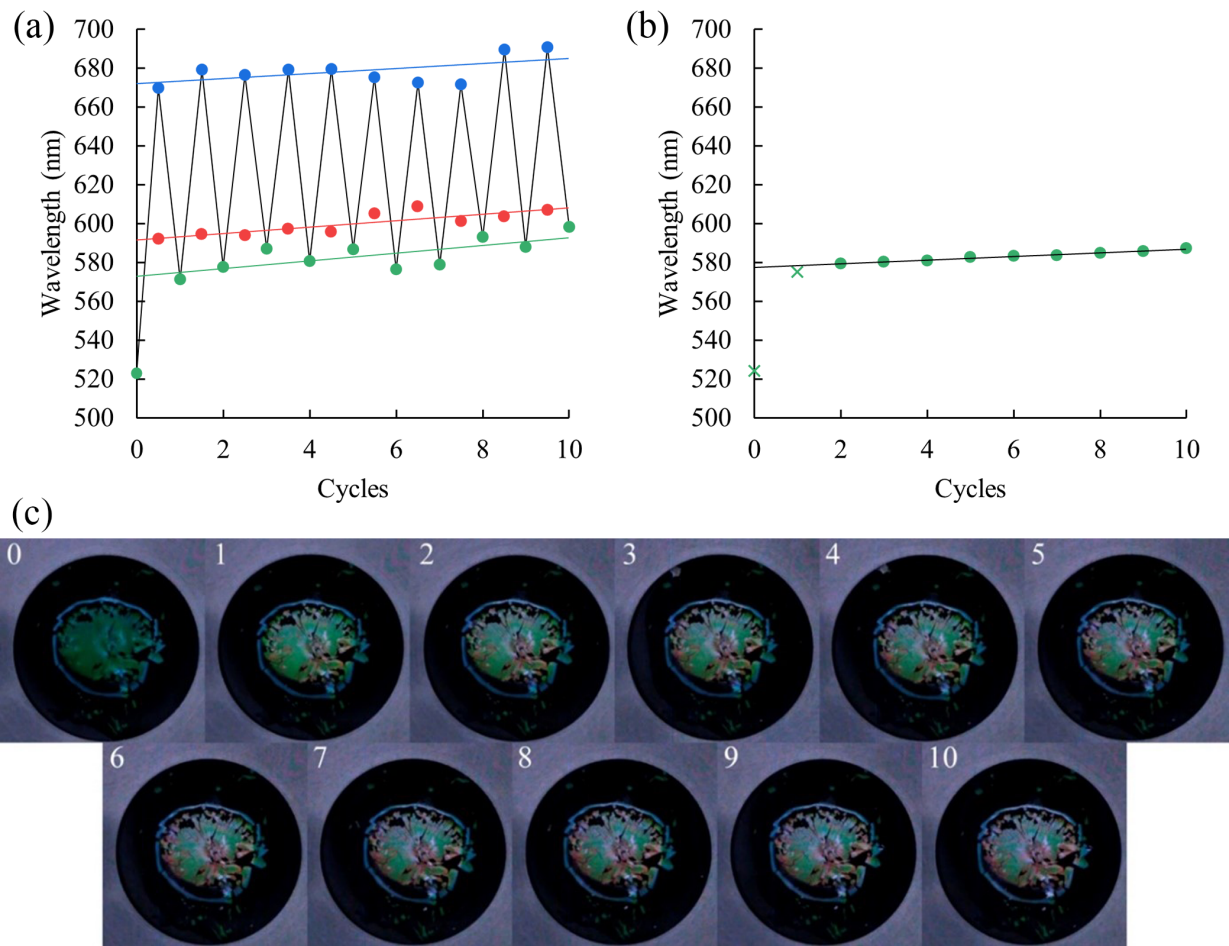
Reversibility of the response to toluene vapor: (a) position of the reflection maximum: green – before exposure to toluene vapor, red – at the response point, blue – 8 min after the exposure (data obtained with an “eye-one Pro” mini-spectrophotometer). (b, c) The starting position of the maximum reflection and initial photo images of the sensor before the next cycle of exposure to toluene vapor (data obtained with a “Ci7800” spectrophotometer).

From the results presented in Figure 7a, we can conclude that there is a wide spread in both the initial position of the PBG and the final one, but this is explained by the heterogeneity of the sensor degradation over the surface area and the small aperture of the mini-spectrophotometer (4.5 mm), which is smaller than the treated sensor area. Before each experiment, the diffuse reflectance spectra were recorded using a device with a larger aperture (10 mm) than the sample size (Figure 7b,c). Even though a partial degradation of the sensor occurs after each detection, it is already linear after the second detection (*R*^2^ = 0.988) and can be taken into account accordingly.

## Conclusion

An approach is proposed for the qualitative determination of aromatic and aliphatic hydrocarbons using stimuli-responsive materials based on 3D photononic crystals. The kinetic regular interactions of organic nonpolar solvents with a photonic crystal-based sensor, having a PDMS sensor matrix, were studied by diffuse reflectance spectroscopy. Vapors of a *p*-xylene/toluene mixture containing the components in different proportions were detected. The possibility of determining the concentration of compounds in a two-component mixture is confirmed.

It was found that after stopping the exposure, the position of the photonic bandgap is almost completely recovered. This fact allows one to perform chemical cycles or online environmental monitoring. However, when exposed to aromatic solvents, sensor degradation is observed, but its linear direction should be noted.

## Experimental

### Materials

Submicrometer particles from linear polystyrene have been synthesized in the Institute of Fine Chemical Technologies RTU MIREA [42]. The sensor matrix has been developed from PDMS “Sylgard 184 silicone elastomer” (Dow Corning, USA). The following organic solvents have been used as analytes: benzene “pur.” and toluene “p.a.” (Reakhim, Russia); *p*-xylene “pur.”, *n*-pentane, “puriss.”, *n*-heptane “puriss. spec.” and *n*-decane “pur.” (EKOS-1, Russia); *n*-octane “pur.” (Acros Organics, Belgium/US).

### Instruments

The average hydrodynamic radius of the PS particles has been determined by using the DLS method on a “Zetasizer Nano ZS” (Malvern Panalytical Ltd, UK) device. Microstructures of sensor matrices have been tested by using the SEM method on an “NVision 40” (Carl Zeiss, Inc., Germany) device and a specular reflectance spectrophotometer “Lambda 950” (PerkinElmer, Inc., USA) in the visible range of the electromagnetic spectrum. The diffuse reflectance spectra have been recorded on a spectrophotometer “Ci7800” (X-Rite, Inc., USA) in the visible range of the electromagnetic spectrum in SCI and SCE modes. The studies of kinetics have been carried out using a mini-spectrophotometer “eye-one Pro” (X-Rite, Inc., USA). The thickness of the stimuli-responsive layer (PDMS and CCA) has been measured by using a “Constant K5” thickness gauge (KONSTANTA LLC, Russia) with an ID2 induction converter. The SEM images have been obtained at the Centre of Shared Equipment of IGIC RAS.

### Sensors

A composite sensor based on 3D PhC, which has a sandwich design, has been used for the experiments. The opal structure is formed on a glass or polymer (polycarbonate or polyethylene terephthalate) substrate. The structure has a close-packed crystal lattice, the nodal points of which contain spherical submicrometer particles of polystyrene with a hydrodynamic diameter of 239.5 nm (polydispersity index 0.101), determined by the DLS method. The CCA has been obtained by self-organization from a water–ethanol suspension [43]. The formed structure was covered with a hydrophobic polymer material layer, that is, polydimethylsiloxane of a specified thickness, which serves as a sensitive layer and mechanically protects the CCA. Sensors with a glass carrier have better optical characteristics but are inferior to polymer regarding material strength.

### Method

**Figure 8:**
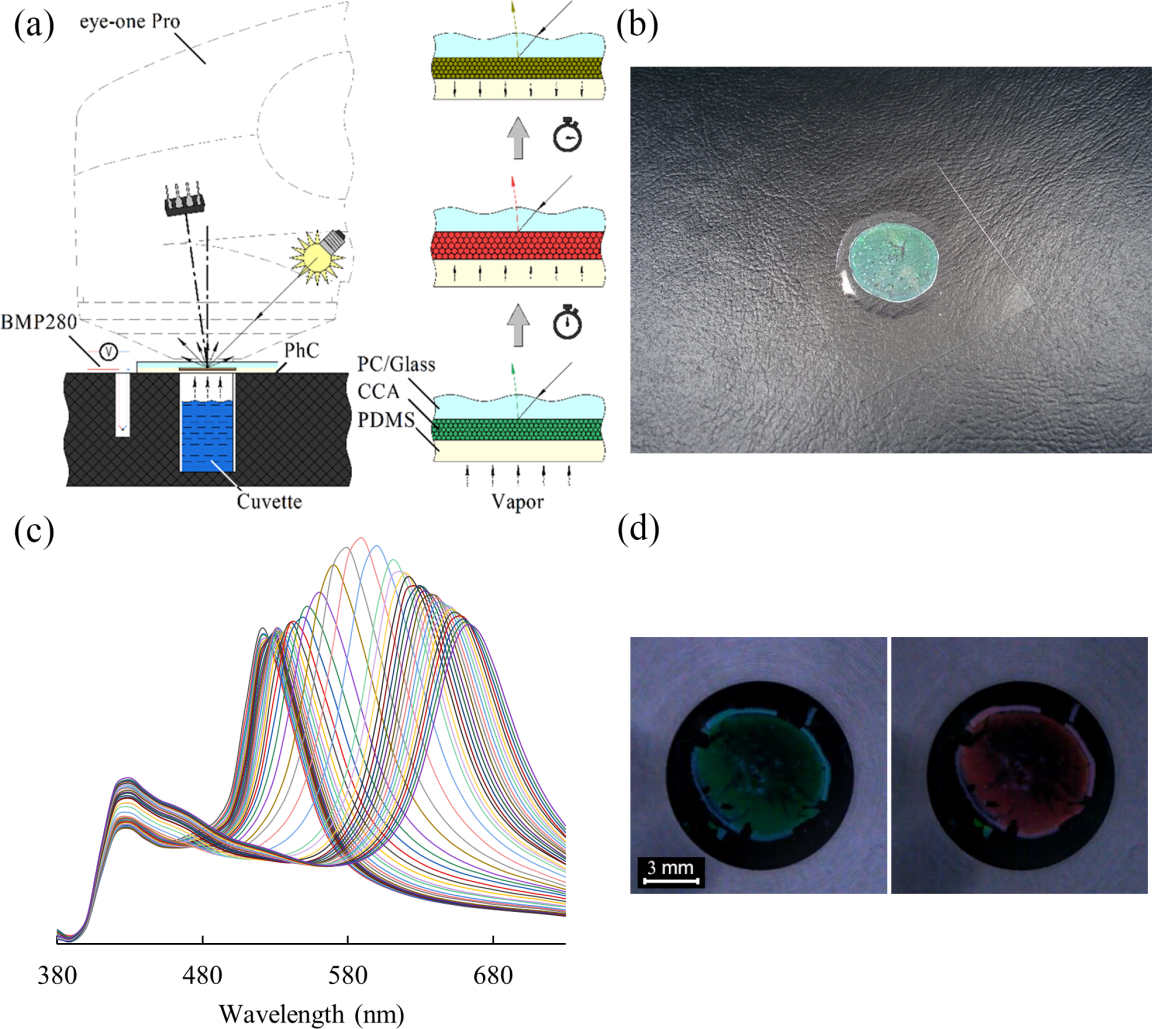
Key points of the experiments: (a) scheme of the experimental equipment; (b) a photo image of the sensor; (c) redshift upon exposure of saturated hydrocarbon vapors on the sensor; (d) photo image of the sensor before and during the exposure.

Figure 8a shows the scheme of the experimental equipment. It consists of the spectrophotometer “eye-one Pro” and a peripheral device, which is made of black composite material based on epoxy resins for visible-light absorption and to elude backward reflection. The diffuse reflectance spectra have been recorded automatically by using the standard software “i1Share v1.4” (X-Rite, Incorporated, USA) and a scripting language program that allowed for receiving data on a preset periodic base. Temperature, pressure and humidity were monitored by a BMP280 sensor (Robert Bosch GmbH, Germany).

A sample of the PhC sensor (Figure 8b) was installed on a cuvette with an analyte solution and fixed with a clamp to avoid vapor leakage during the exposure. The sensitive side of the sample was faced into the cuvette with an analyte and the diffuse reflectance spectra were recorded through the optically transparent carrier. A negligible reflection of the polycarbonate substrate is in the blue spectrum region (below 420 nm) and does not overlap with the reflection of the photonic crystal. As a result of the experiment, we obtained spectra measured at a predetermined time interval (Figure 8c). The color of the sensor changed from green to red (Figure 8d).

## References

[1] Wang Z, Zhang J, Li J, Xie J, Li Y, Liang S, Tian Z, Li C, Wang Z, Wang T (2011). J Mater Chem.

[2] Smith N L, Hong Z, Asher S A (2014). Analyst.

[3] Elsherif M, Hassan M U, Yetisen A K, Butt H (2018). ACS Nano.

[4] Lova P, Comoretto D (2018). AIP Conf Proc.

[5] Chen C, Dong Z-Q, Shen J-H, Chen H-W, Zhu Y-H, Zhu Z-G (2018). ACS Omega.

[6] Qi F, Yan C, Meng Z, Li S, Xu J, Hu X, Xue M (2019). Anal Bioanal Chem.

[7] Burratti L, Casalboni M, De Matteis F, Pizzoferrato R, Prosposito P (2018). Materials.

[8] Sato A, Ikeda Y, Yamaguchi K, Vohra V (2018). Nanomaterials.

[9] Burratti L, De Matteis F, Casalboni M, Francini R, Pizzoferrato R, Prosposito P (2018). Mater Chem Phys.

[10] Li G, Xiao F, Liao S, Chen Q, Zhou J, Wu Z, Yu R (2018). Sens Actuators, B.

[11] Hong W, Li H, Hu X, Zhao B, Zhang F, Zhang D (2012). Chem Commun.

[12] Yuan Y, Li Z, Liu Y, Gao J, Pan Z, Liu Y (2012). Chem – Eur J.

[13] Zhang Y-H, Ren H-H, Yu L-P (2018). Anal Methods.

[14] Busch K, John S (1998). Phys Rev E.

[15] Wang F, Zhu Z, Xue M, Xue F, Wang Q, Meng Z, Lu W, Chen W, Qi F, Yan Z (2015). Sens Actuators, B.

[16] Kuo W-K, Weng H-P, Hsu J-J, Yu H H (2016). Mater Chem Phys.

[17] Baca J T, Finegold D N, Asher S A (2008). Analyst.

[18] Arunbabu D, Sannigrahi A, Jana T (2011). Soft Matter.

[19] Lin F Y, Yu L P (2012). Anal Methods.

[20] Ruan J-L, Chen C, Shen J-H, Zhao X-L, Qian S-H, Zhu Z-G (2017). Polymers (Basel, Switz).

[21] Zeng F, Wu S, Sun Z, Xi H, Li R, Hou Z (2002). Sens Actuators, B.

[22] Sharma A C, Jana T, Kesavamoorthy R, Shi L, Virji M A, Finegold D N, Asher S A (2004). J Am Chem Soc.

[23] Walker J P, Asher S A (2005). Anal Chem (Washington, DC, U S).

[24] Xiao F, Li G, Wu Y, Chen Q, Wu Z, Yu R (2016). Anal Chem (Washington, DC, U S).

[25] Song Y, Bai J, Zhang R, He H, Li C, Wang J, Li S, Peng Y, Ning B, Wang M (2018). Anal Chem (Washington, DC, U S).

[26] Yan C, Qi F, Li S, Xu J, Liu C, Meng Z, Qiu L, Xue M, Lu W, Yan Z (2016). Talanta.

[27] Xu J, Yan C, Liu C, Zhou C, Hu X, Qi F (2017). IOP Conf Ser: Mater Sci Eng.

[28] Guo C, Zhou C, Sai N, Ning B, Liu M, Chen H, Gao Z (2012). Sens Actuators, B.

[29] Sai N, Ning B, Huang G, Wu Y, Zhou Z, Peng Y, Bai J, Yu G, Gao Z (2013). Analyst.

[30] Matsuguchi M, Uno T (2006). Sens Actuators, B.

[31] Alizadeh T, Rezaloo F (2013). Int J Environ Anal Chem.

[32] Shim D-Y, Chang S-M, Kim J M (2021). Sens Actuators, B.

[33] Bol’shakov E S, Ivanov A V, Kozlov A A, Abdullaev S D (2018). Russ J Phys Chem A.

[34] Woodcock L V (1997). Nature.

[35] Abrarov S M, Kim T W, Kang T W (2006). Opt Commun.

[36] Fudouzi H (2004). J Colloid Interface Sci.

[37] Gajiev G M, Golubev V G, Kurdyukov D A, Medvedev A V, Pevtsov A B, Sel’kin A V, Travnikov V V (2005). Phys Rev B.

[38] Sinitskii A S, Khokhlov P E, Abramova V V, Laptinskaya T V, Tretyakov Y D (2007). Mendeleev Commun.

[39] Schneider F, Draheim J, Kamberger R, Wallrabe U (2009). Sens Actuators, A.

[40] Sultanova N, Kasarova S, Nikolov I (2009). Acta Phys Pol, A.

[41] Rumens C V, Ziai M A, Belsey K E, Batchelor J C, Holder S J (2015). J Mater Chem C.

[42] Shragin D I, Gritskova I A, Kopylov V V, Milushkova E V, Zlydneva L A, Levachev S M (2015). Silicon.

[43] Ivanov A V, Kozlov A A, Koreshkova A N, Abdullaev S D, Fedorova I A (2017). Moscow Univ Chem Bull (Engl Transl).

